# The Benefits of Plant Extracts for Human Health

**DOI:** 10.3390/foods9111653

**Published:** 2020-11-12

**Authors:** Charalampos Proestos

**Affiliations:** Laboratory of Food Chemistry, Department of Chemistry, National and Kapodistrian University of Athens, 15771 Athens, Greece; harpro@chem.uoa.gr; Tel.: +30-210-727-4160

Nature has always been, and still is, a source of foods and ingredients that are beneficial to human health. Nowadays, plant extracts are increasingly becoming important additives in the food industry due to their content in bioactive compounds such as polyphenols [1] and carotenoids [2], which have antimicrobial and antioxidant activity, especially against low-density lipoprotein (LDL) and deoxyribonucleic acid (DNA) oxidative changes [3]. The aforementioned compounds also delay the development of off-flavors and improve the shelf life and color stability of food products. Due to their natural origin, they are excellent candidates to replace synthetic compounds, which are generally considered to have toxicological and carcinogenic effects. The efficient extraction of these compounds from their natural sources and the determination of their activity in commercialized products have been great challenges for researchers and food chain contributors to develop products with positive effects on human health. The objective of this Special Issue is to highlight the existing evidence regarding the various potential benefits of the consumption of plant extracts and plant extract-based products, along with essential oils that are derived from plants also and emphasize in vivo works and epidemiological studies, application of plant extracts to improve shelf-life, the nutritional and health-related properties of foods, and the extraction techniques that can be used to obtain bioactive compounds from plant extracts. 

In this context, Concha-Meyer et al. [4] studied the bioactive compounds of tomato pomace obtained by ultrasound assisted extraction. In this review, it was presented that the functional extract obtained by ultrasounds had antithrombotic properties, such as platelet anti-aggregant activity compared with commercial cardioprotective products. Turrini et al. [5] introduced bud-derivatives from eight different plant species as a new category of botanicals containing polyphenols and studied how different extraction processes can affect their composition. Woody vine plants from Kadsura spp. belonging to the Schisandraceae family produce edible red fruits that are rich in nutrients and antioxidant compounds such as flavonoids. Extracts from these plants had antioxidant properties and had shown also key enzyme inhibitions [6]. Hence, fruit parts other than the edible mesocarp could be utilized for future food applications using Kadsura spp. rather than these being wasted. Saji et al. [7] studied the possible use of rice bran, a by-product generated during the rice milling process, normally used in animal feed or discarded due to its rancidity, for its phenolic content. It was proved that rice bran phenolic extracts via their metal chelating properties and free radical scavenging activity, target pathways of oxidative stress and inflammation resulting in the alleviation of vascular inflammatory mediators. Villedieu-Percheron et al. [8] evaluated three natural diterpenes compounds extracted and isolated from *Andrographis paniculata* medicinal herb as possible inhibitors of NFκB (nuclear factor kappa-light-chain-enhancer of activated B cells) transcriptional activity of pure analogues. Yeon et al. [9] evaluated the antioxidant activity, the angiotensin I-converting enzyme (ACE) inhibition effect, and the α-amylase and α-glucosidase inhibition activities of hot pepper water extracts both before and after their fermentation. These water extracts were proved to have potentially inhibitory effects against both hyperglycemia and hypertension. The hydrolyzed extracts of Ziziphus jujube fruit, commonly called jujube, were examined for their protective effect against lung inflammation in mice [10]. They contained significant amounts of flavonoids which inhibited cytokine release from macrophages and promoted antioxidant defenses in vivo. Tran at al. [11] examined the antidiabetic activity of spray-dried Euphorbia hirta L. herb extracts containing high concentrations of bioactive compounds such as phenolics and flavonoids. Li et al. [12] reported that intestinal microbiota is closely associated with the initiation and progression of diabetes mellitus and reviewed bioactive components which exhibited anti-diabetic activity by modulating these intestinal microbiotas. Essential oils have promising activity against antibiotic-resistant bacteria and chemotherapeutic-resistant tumors. This was supported by the study of Viktorová et al. [13] where lemongrass essential oil and especially citral, the dominant component, proved to have potential antimicrobial and anticancer activity. Additionally, Mitropoulou et al. [14] investigated the antimicrobial potential of *Sideritis raeseri subps. raeseri* essential oil against common food spoilage and pathogenic microorganisms and evaluated its antioxidant and antiproliferative activity. Salehi et al. [15] reviewed the Berberis plants, which contain alkaloids, tannins, phenolic compounds and essential oils, and their possible use in the food and pharmaceutical industry. Last but not least, Kiokias et al. [16] reviewed the naturally occurring phenolic acids from plants and their antioxidant activities in o/w emulsions and in vitro lipid-based model systems.

Still more research is needed to explore more and in depth the health beneficial effects of plant extracts, since nature certainly has more to give to humans.
